# Intertidal Warming Causes Mortality and Disrupts the Microbiome of Oysters

**DOI:** 10.1111/1462-2920.70152

**Published:** 2025-07-18

**Authors:** Elliot Scanes, Nachshon Siboni, Maquel Brandimarti, Justin Seymour

**Affiliations:** ^1^ Climate Change Cluster University of Technology Sydney Ultimo New South Wales Australia; ^2^ Parks Bay Marine Lab Koolewong New South Wales Australia

## Abstract

Intertidal ecosystems are physically stressful habitats, with resident organisms often living close to their limits. These limits include the balance between host organisms and microbial partners; a balance that may be tipped by climate change. We simulated intertidal warming in the field by establishing populations of the Sydney rock oyster, *Saccostrea glomerata,* on black and white concrete tiles, resulting in differing thermal conditions. Tiles were placed on the intertidal shoreline among natural oyster populations. Oysters on black tiles were up to 3°C warmer than those on white tiles during low tide. We monitored the tiles for oyster survival and took gill and haemolymph samples from oysters for microbiological analysis using qPCR, 16S, and HSP60 rRNA sequencing. We found that after six days, levels of oyster mortality were 50% greater on the black tiles. Oysters on black tiles exhibited a significant shift in their microbiome, involving increases in putative pathogenic bacteria from the *Vibrio* genus, including the known oyster pathogen *V. harveyi* and the human pathogen *V. parahaemolyticus* . These findings demonstrate that relatively small increases in temperature within intertidal ecosystems can cause significant shifts in the microbiome and mortality among oyster populations, with putative links to bacterial pathogens.

## Graphical abstract

We used black and white coloured tiles to manipulate intertidal oyster temperature. Oysters on black tiles experienced a shift in their microbiome compared to oysters on white tiles, with a significant increase in bacteria from the *Vibrio* genus, including putative oyster (e.g., *V. harveyi* ) and human (e.g., *V. parahaemolyticus* ) pathogens.

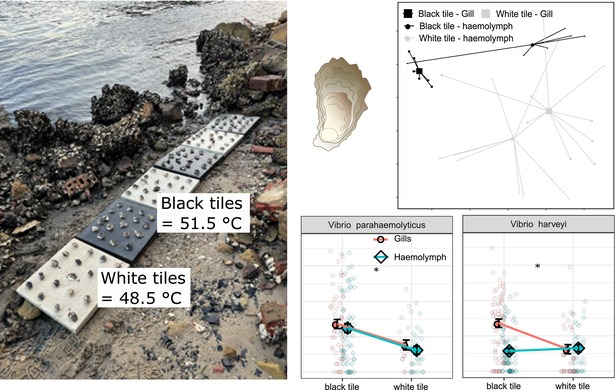

## 1 Introduction

The intertidal marine environment is among the most physically stressful on earth, with many species residing close to their physiological limit, making intertidal habitats highly vulnerable to climate change (Helmuth, Mieszkowska, et al. 2006; Scanes et al. 2017). Intertidal ecosystems are typically dominated by marine flora and fauna that can endure periods of emersion, creating an environment devoid of shade except behind boulders and in crevices (Bauer et al. 2024). Sessile organisms like oysters and barnacles cannot move towards shade and therefore must endure direct solar radiation when low tides coincide with the hottest part of the day, raising their body temperature well above the air temperature (Helmuth, Mieszkowska, et al. 2006; White et al. 2023). Living close to their physiological limits means that an increase in temperatures can have catastrophic outcomes for intertidal organisms (Helmuth, Mieszkowska, et al. 2006). For example, when an atmospheric heatwave coincided with low tides in the Pacific Northwest of America in 2021, up to 70% of sessile marine invertebrates died, with estimates of over 1 million dead mussels in a 100 m stretch of shoreline (White et al. 2023). During this heatwave, surface temperatures of 50°C were recorded in the intertidal zone of the Salish Sea. It is anticipated that climate change and the resulting heatwaves will make life more challenging for sessile invertebrates in already hostile rocky intertidal habitats (Helmuth, Mieszkowska, et al. 2006; Hesketh and Harley 2023).

Intertidal habitats are one of the few habitats exposed to both atmospheric and marine extreme events (Helmuth, Mieszkowska, et al. 2006). Marine heatwaves can impact marine communities across all trophic levels (Wernberg et al. 2023, 2013). Numerous studies have demonstrated the detrimental impacts of marine heatwaves, such as on the Western Australian coastline, where a marine heatwave altered the biodiversity of temperate seaweeds, sessile invertebrates, and demersal fish and led to a reduction in the abundance of habitat‐forming seaweeds (Wernberg et al. 2013). The effects of marine and atmospheric heatwaves have been observed in the intertidal zone, where impacts on habitat‐forming organisms can have dramatic flow‐on effects throughout the ecosystem (Wernberg et al. 2023). For example, heatwaves and low rainfall in 2015–2016 caused widespread mortality of habitat‐forming mangroves over 1000kms of Northern Australian coastline, resulting in immediate consequences for ecosystem services including multi‐million dollar (AUD) fisheries, carbon capture, and shoreline protection (Duke et al. 2017; Sippo et al. 2018).

There is also evidence that heatwave conditions can profoundly reshape the important interactions between marine animals and their microbiome (Tout et al. 2015; Green et al. 2019; Scanes et al. 2023). Evidence from coral reefs demonstrates that marine heatwaves can alter the microbiome of the coral hosts with negative consequences for coral fitness (Vompe et al. 2024; Rubio‐Portillo et al. 2016; Voolstra et al. 2024). Similar observations have been made of habitat‐forming seaweeds, with heatwaves triggering disease and even increases in predation caused by changes in the microbiome (Qiu et al. 2019; Castro et al. 2024). Another habitat‐forming organism vulnerable to bacterial disease is oysters.

Oysters are important habitat forming organisms that inhabit intertidal and coastal zones around the world (Grabowski et al. 2012; Grabowski and Peterson 2007), but are vulnerable to both atmospheric and marine heatwaves (Siboni et al. 2024; Scanes, Parker, et al. 2020; Xu et al. 2021; Scanes et al. 2025), with bacterial disease a leading cause of mortality (Scanes et al. 2023; Siboni et al. 2024; Petton et al. 2019). Research has demonstrated that a compromised immune system resulting from warming or viral infection permits bacteria, often from the *Vibrio* genus, to infect and kill oysters (Scanes et al. 2023; Siboni et al. 2024; De Lorgeril et al. 2018; Wendling et al. 2017; Montánchez and Kaberdin 2020). Bacteria from the *Vibrio* genus are naturally occurring in marine and coastal habitats, with many members identified as potentially pathogenic to marine organisms and human seafood consumers (Ndraha et al. 2020). *Vibrio* are also identified as a marine pathogen predicted to increase under climate change because they often proliferate in warmer conditions (Scanes et al. 2025; Baker et al. 2022; Baker‐Austin et al. 2013; Mora et al. 2022).

Following exposure to both atmospheric and marine heatwave conditions, multiple species of oysters have displayed significant microbiome shifts, which often involve substantial increases in *Vibrio*, most notably putative pathogens from the *Vibrio harveyi* clade (Green et al. 2019; Scanes et al. 2023). These changes in the oyster microbiome were also accompanied by high levels of oyster mortality and a decrease in circulating white blood cells (Scanes et al. 2023). Oyster microbiomes are not only a source of disease, but are vital to providing immune processes to the host (King, Jenkins, Seymour, et al. 2019) and aiding in digestion (Pimentel et al. 2021). Oysters provide irreplaceable habitat in intertidal zones, but are also a valuable aquaculture commodity, with more than 6.2 million tonnes produced each year around the globe (Food and Agriculture Organization 2020). Climate change, therefore, has the potential to disrupt global ecosystems and food security by altering oyster microbiomes and increasing disease.

The scale and complexity of the climate change process create challenges when designing experiments to simulate climate change processes (Boyd et al. 2018; Hemraj and Russell 2025). Laboratory‐based climate change experiments allow for a high degree of control (Green et al. 2019; Scanes et al. 2023; Petton et al. 2019), but cannot account for natural variability and ecological interactions, providing constraints on their capacity to predict the responses of marine organisms to climate change (Boyd et al. 2018). In contrast, an increasing number of experiments have made use of already warming habitats to observe responses by marine organisms (Wernberg et al. 2013; Siboni et al. 2024; King, Jenkins, Go, et al. 2019), but this approach restricts the ability to compare precisely controlled treatments. In order to advance our understanding of climate change impacts on important marine species, experiments that incorporate natural variability in real‐world scenarios while also retaining control of experimental variables are needed (Boyd et al. 2018).

Understating the effect heatwaves have on the interactions between intertidal aquatic organisms and their microbiome will be essential to inform how species will respond to climate change. Eastern Australia is an ocean warming hotspot, where sea‐surface temperatures are warming at twice the rate of the global average (Hobday and Pecl 2014). However, Australia's land temperatures are warming 40% faster than the oceans (Bureau of Meteorology 2018). This simultaneous warming from both land and sea is putting significant pressure on Australian intertidal rocky shore communities. This study aimed to use a simple, but novel manipulative field experiment to test the effects of intertidal warming on the microbiome and survival of oysters. We used a passive heating design with light and dark coloured tiles to manipulate temperatures in the field. We hypothesised that elevated temperatures would result in shifts in the oyster bacterial communities, underpinned by an increase in putative pathogens, and ultimately an increase in oyster mortality.

## 2 Experimental Procedures

### 2.1 Experimental Design and Sampling Protocols

All experiments were conducted within the natural intertidal shoreline at a site on the east coast of Australia (Brisbane Water, NSW, −33.4773, 151.3221) between 30/1/24 and 6/2/24. This region is characterised by a warm temperate/subtropical climate and supports an oyster aquaculture industry, which primarily targets the Sydney rock oyster, *Saccostrea glomerata*. The catchment of Brisbane Water is approximately 45% urbanised. Adjacent to the study site was a community of seagrass (*Zostera mulleri*), and during the experiment, juvenile fish including *Acanthopagrus australis, Mugil cephalus*, and the estuary stingray *Dasyatis fluviorum* were observed to inhabit the study site when immersed.

To experimentally manipulate the temperatures experienced by oysters within the natural inter‐tidal setting, we used a novel approach involving attaching oysters to concrete tiles (400 × 400 × 40 mm) to generate a thermal gradient (McAfee et al. 2017). For these experiments, two tile types were used, which were identical in composition but varied in colour, with dark grey/black and cream/white colour tiles used. Pilot studies demonstrated that when left in the direct sun, the surface of the darker tiles heated to temperatures 3°C–4°C greater than the white tiles.

**FIGURE 1:**
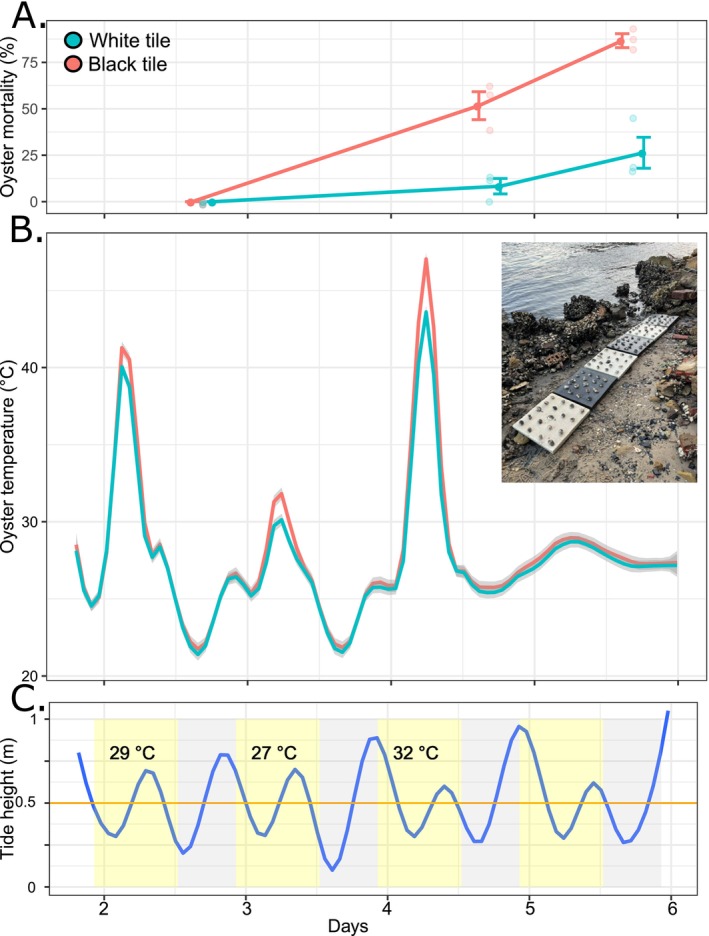
(A) Mean (±SE) oyster mortality (%) over the experimental period. Tile colours are indicated by colours whereas black tiles in red and white tiles in turquoise. Small points indicate raw data. (B) Mean oyster temperature (°C) recorded on the tiles, shaded area indicates 95% confidence intervals. Picture insert is a photo of the experimental setup. (C) Tidal heights (m) over the experimental period, grey shaded areas indicate night time and yellow shading indicates daytime, text indicates maximum daytime temperatures recorded by the Australian Bureau of Meteorology at the nearest weather station (10 km distance).

To simulate a population of oysters inhabiting the intertidal shore, 16 Sydney rock oysters (*Saccostrea glomerata*) were glued to the upper side of each concrete tile. Oysters were obtained from existing farming stock of an oyster grower in Brisbane Waters (Empire Bay Oysters), were 13 months old and had a mean (±SE) shell length of 25 ± 6.2 mm. The experimental oysters were genetically “wild” and had previously been caught on sticks in an estuary 300 km north of our study location. Prior to collection from the farmer, oysters were kept on intertidal tray leases. Oysters were glued to the tiles using construction adhesive (Sika Co.) at an even spacing of 60 mm apart (Figure 1). To continuously log the temperature experienced by oysters during experiments, thermochron temperature loggers (ibutton DS1921G; ±0.5°C) were placed inside an empty oyster shell and glued onto each tile alongside the live oysters. To do this, an oyster from the same source as live oysters was shucked and the tissue was removed. The shell was then filled with epoxy resin (3 M Scotch‐cast) with thermal properties comparable to those of oyster tissue (McAfee et al. 2017). The temperature logger was placed inside the shell, covered in resin and then the upper valve was replaced onto the shell. The logger and shell were then glued to the tile in the same way as live oysters. To understand how the temperatures on tiles related to the natural environment, three loggers in oyster shells were also glued imbedded among the natural *S. glomerata* aggregations at 0.5 m LAT.

Six tiles were then organised in alternating colours (white, black, white…) in a single row parallel to the shoreline. Tiles were organised on a patch of sand among sandstone boulders that were covered in natural aggregations of *S. glomerata* at heights on the shore from 0.2 to 0.8 m above Lowest Astronomical Tide (LAT). Tiles were placed at 0.5 m above LAT to ensure immersion during high tide. Tiles remained positioned there for 5 days, after which they were moved to 0.3 m below LAT following hot weather to allow for recovery and to monitor survival. The tiles then remained in place for six days, coinciding with low tides occurring around midday.

Tiles were checked daily for mortality, with oysters determined as dead when the upper valve no longer remained closed. After six days, tiles were collected and three live oysters from each tile were randomly sampled. Oysters were shucked while still attached to the tile and 0.2 mL of haemolymph was extracted from the pericardial cavity using a sterile needled syringe, placed into a cryovial and then frozen in liquid Nitrogen (LN_2_). Gill tissue was then excised using sterile scissors, placed in a cryovial and frozen in LN_2_. White tiles were re‐deployed at 0.3 m LAT for a further seven days to monitor oyster survival, but at this point of the experiment there were no remaining live oysters on the black tiles.

On the day oyster tissue was sampled, triplicate seawater samples were also taken from the surface seawater directly adjacent to the tiles on the shore. Seawater samples were collected at high tide in water of 1 m depth. Samples were collected in three 10 L pre‐cleaned containers (rinsed with 10% bleach, washed three times with water and washed three times with seawater prior to sampling) and filtered on site through 0.22 μm pore‐size membrane filters (Millipore, Durapore PVDF) using a peristaltic pump (100 rpm). Filter membranes were immediately placed into cryovials and frozen in LN_2_.

### 2.2 Molecular Microbiological Analysis

DNA was extracted from oyster tissue samples using the QIAGEN DNeasy Blood and Tissue kit, while DNA from seawater was extracted using the QIAGEN DNeasy Power Water kit. DNA was stored at −80°C until further processing.

To characterise bacterial community composition, PacBio full length 16S rRNA amplicon sequencing was used. DNA was amplified using the 27F 5′‐AGRGTTYGATYMTGGCTCAG‐3′ and 1492R 5′‐RGYTACCTTGTTACGACTT‐3′ primer pairing, with sequencing performed at the Australian Genome Research Facility (AGRF, Melbourne, Australia). Raw data files in FASTQ format were deposited to the Sequence Read Archive (SRA) under Bioproject number [PRJNA1281817; http://www.ncbi.nlm.nih.gov/bioproject/1281817].

Raw demultiplexed 16S rRNA data was processed using the Quantitative Insights into Microbial Ecology (QIIME 2 version 2019.1.0) pipeline. Briefly, single‐end read sequences were imported, trimmed, and denoised using DADA2 (version 2019.1.0). Sequences were identified at the single nucleotide level (Amplicon Sequence Variants; ASV) and taxonomy was assigned using the classify‐sklearn qiime feature classifier against the Silva v138.1 database. Unassigned ASVs, or those assigned as mitochondria or chloroplast, as well as rare reads (below relative abundances of 0.001%) were filtered out. Rarefaction plots were used to check sequencing depth, and data were rarefied to 7000 sequences per sample, which excluded four samples (3 gills, 1 hemolymph) that were not used for further analysis.

Vibrio are known pathogens of oysters and other marine animals (King, Jenkins, Seymour, et al. 2019), but are not well characterised by the 16S gene. To characterise the *Vibrio* community, we used a custom amplicon sequencing assay targeting the hsp60 gene (King, Siboni, et al. 2019). Raw demultiplexed hsp60 data was processed as previously described (King, Siboni, et al. 2019). Reads were joined using Flash (Magoč and Salzberg 2011) and the resulting fragments were trimmed with Mothur (Schloss et al. 2009). Fragments were clustered into operational taxonomic units (OTUs) at the 97% threshold and chimeric sequences were removed using vsearch (Rognes et al. 2016). Taxonomy was assigned to fragments using QIIME (Caporaso et al. 2010) and the RDP classifier (Wang et al. 2007) according to a custom *Vibrio*‐hsp60 reference dataset (King et al. 2019). OTUs with less than 25 total reads were removed and sequences were normalised to the number of sequences per sample to produce relative abundance.

Measurements of total bacterial, *Vibrio* and *V. harveyi* abundances were determined by quantitative PCR (qPCR). Abundances of total bacteria were quantified by targeting the bacterial 16S rRNA gene, using the BACT1369F and PROK1492R primer pair and the TM1389F probe (Suzuki et al. 2000). Abundances of total *Vibrio* were quantified with the primer pair Vib1‐f and Vib2‐r (Thompson et al. 2004; Siboni et al. 2016), which targets the 16S rRNA gene specific to the *Vibrio* genus. The putative pathogen *Vibrio harveyi* has consistently been implicated in oyster mortality events, including in *S. glomerata*, following heatwaves (Green et al. 2019; Scanes et al. 2023). To quantify the abundances of *V. harveyi* , we used the primer pair of mreB11F and mreB9bisR (Mougin et al. 2020), which targets the protein (MreB) that has previously been aligned to 171 *Vibrio harveyi* strains from NCBI. We then constructed a TaqMan probe 5′‐FAM‐AACTACGGCAGCTTGATCGGTGAA‐ZEN‐IBFQ‐3′ on a conserved area between the two primers.

Quantitative PCR (qPCR) assays were prepared with an epMotion 5075I Automated Liquid Handling System and performed on a Bio‐Rad CFX384 Touch Real‐Time PCR Detection System, with three technical replicates, a standard curve, and negative controls. qPCR protocols for the assays followed those used in Scanes et al. (2023). The resulting data were normalised to gene copies per litre of seawater and per mg of haemolymph or gill tissue.

### 2.3 Data Analysis

Comparison of oyster survival between tile type and days was performed using a two‐way ANOVA, with “Tile colour” and “Time” as the factors. Pairwise Tukey Tests were used to determine the source of significant variation (*n* = 9). Differences in bacterial alpha diversity metrics, derived from both 16S rRNA and hsp60 sequencing data, between tile colours were determined using a single factor ANOVA (*n* = 9), while differences in bacterial beta diversity were assessed using PERMOVA (Vegan package (Dixon 2003; Oksanen et al. 2007)) on Weighted‐unifrac (16S rRNA) or Bray–Curtis (hsp60) distance matrices with “Tile colour” and “Tissue type” (haemolymph vs. gill) as the factors. ANCOM‐bc2 analysis was used to determine significant differences in the relative abundance of ASVs between tile colours for both tissue types, using a Benjamin–Hochberg adjusted *p* value set at < 0.05. Differences in bacterial abundance from qPCR were determined using two‐way ANOVA with “Tissue type” and “Tile colour” as the fixed factors and “Replicate tile” as a random factor. Differences in the relative abundance of vibrio OTUs assigned to the same species were determined using a generalised linear mixed model (GLMM; MASS package (Ripley et al. 2013)) with a negative binomial distribution and the factors “Tissue type”, “Tile colour” as fixed factors and “Replicate tile” as a random factor. GLMM *p* values were adjusted for multiple comparisons using a Bonferroni correction.

## 3 Results

### 3.1 Temperature Treatments

Oysters on the white tiles experienced a mean (±SE) temperature of 27.07°C ± 0.07°C and a maximum of 48.5°C, while oysters on black tiles experienced a mean (±SE) temperature of 27.4°C ± 0.08°C and a maximum of 51.5°C. The black tile treatment increased the mean temperature of oysters by 0.38°C ± 0.11°C (mean ± SE, *n* = 3) over the experimental period. The temperature of oysters on the black tiles exceeded that of the white tiles for 74 ± 24 h (mean ± SE, *n* = 3), which represented 51% of the experiment duration. These periods of elevated temperature on the black tiles all corresponded with emersion at low tide (Figure 1). The maximum temperature difference between white and black tile treatments in any replicate was 3°C, and the mean maximum difference in temperature across the three replicates was 2.8°C ± 0.16°C (mean ± SE, *n* = 3). The mean (±SE) temperature of wild oysters was 27.3°C ± 0.09°C, reaching a maximum temperature of 45°C.

### 3.2 Oyster Survival

The elevated temperatures experienced on black tiles significantly increased the mortality of oysters (ANOVA Colour × Time; F_1,14_ = 34.1, *p* < 0.001). For the first three days of the experiment, there was no mortality recorded on either treatment type. On day four, there was no mortality recorded, but oysters on the black tiles began to show signs of shell gaping and a slowed response to close their shell. On day five, however, mortality was recorded on both white and black tiles, but mortality on black tiles was significantly (Pairwise test; *p* < 0.001) greater, whereby mean mortality reached 51% ± 7.5% (±SE) compared to only 8.3% ± 4.1% on white tiles. On day 6, there were again significant differences between treatments (Pairwise test; *p* < 0.001), with mean mortality reaching 86% ± 3.7% (±SE) on black tiles, compared to 26% ± 8.3% on white tiles (Figure 1).

### 3.3 Bacterial Concentrations

**FIGURE 2:**
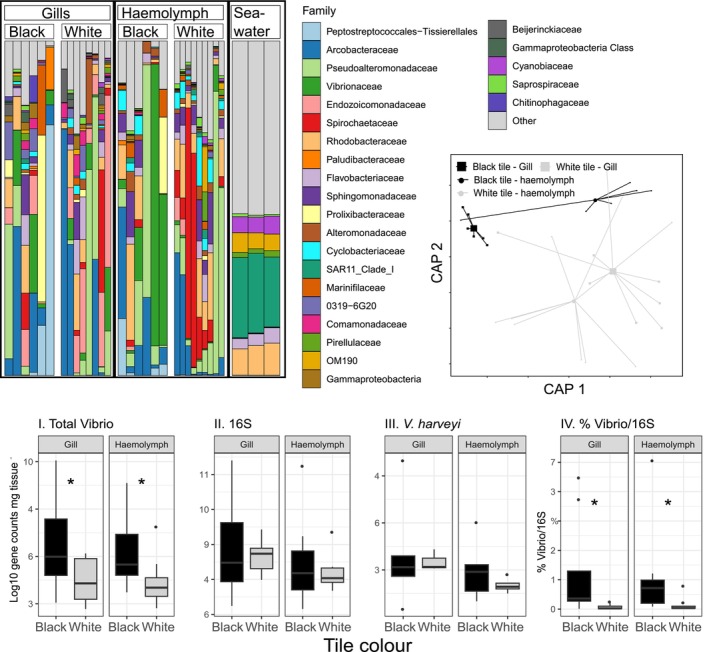
(A) Stacked bar plot indicating the relative abundance of bacteria at the family level in oyster gill and haemolymph tissue as well as seawater samples. (B) Constrained Analysis of Principal Coordinates (CAP) ordination plot. Oyster tissues and tile colour are indicated by shapes and line colour. (C) bacterial concentrations as determined by qPCR for (I) *Vibrio*, (II) 16S, (III) *V. harveyi* and (IV) the % of Vibrio within the bacterial community (vibrio/16S).

While no significant differences in the total abundance of bacteria, defined using qPCR targeting the bacterial 16S rRNA gene, were observed between treatments, elevated temperatures on black tiles significantly increased the concentration of bacteria from the *Vibrio* genus (Figure 2). Both gill and haemolymph samples from oysters located on black tiles contained significantly more *Vibrio* than oysters on white tiles (ANOVA F_1,27_ = 8.6, *p* < 0.05). Furthermore, when the abundance of *Vibrio* was normalised to total bacterial abundance (% *Vibrio* / 16S), the oysters on black tiles had a significantly greater proportion of their population comprised of *Vibrio* in both gill (mean ± SE; Black = 0.9% ± 0.5%, White = 0.1% ± 0.08%) and haemolymph (mean ± SE; Black = 1.1% ± 0.5%, White = 0.07% ± 0.03%) tissues (ANOVA F_1,26_ = 6.1, *p* < 0.05). There were no significant differences in *V. harveyi* abundance.

Seawater samples contained both *Vibrio* and *V. harveyi* . The mean concentration of *Vibrio* in seawater was 7 × 10^5^ ± 34,941 gene copies L^−1^ (mean ± SE), while *V. harveyi* was 3798 ± 1103 gene copies L^−1^.

### 3.4 Bacterial Community Composition

Elevated temperatures on black tiles significantly altered the bacterial communities within oyster gills and haemolymph (Figure 2). Bacterial communities from the haemolymph of oysters on black tiles had a significantly greater Chao1 index of diversity (ANOVA, F_1,13_ = 4.8, *p* < 0.05) than those on white tiles. However, no significant difference in Chao1 occurred in bacterial communities in oyster gills (ANOVA *p* > 0.05). There were no significant differences observed between tile colour treatments for the bacterial communities in either oyster haemolymph or gills for the Simpsons or Shannon's indices of diversity (*p* < 0.05).

**FIGURE 3:**
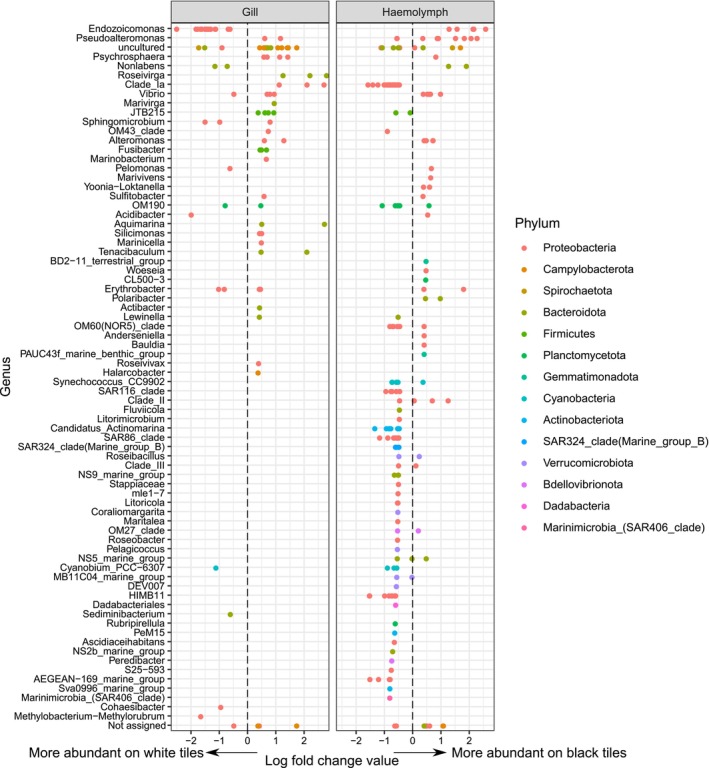
Amplicon Sequence Variants (ASVs) from oyster tissue that were significantly different in abundance on black compared to white tiles. Positive log fold change values indicate a greater abundance on black tiles. Bacterial phylum are indicated by colour. ASVs with a log fold change value between −0.4 and 0.4 have been removed for clarity. Significance was determined using ANCOM‐BC2 analysis.

There were significantly different bacterial communities on black compared to white tiles for both oyster haemolymph and gill tissues (PERMAOVA tile colour × sample type; F_1,28_ = 1.4, *p* < 0.05). Ordination plots showed the bacterial communities from our two temperature treatments to be separated, although there was greater variability among oysters on white tiles. Bacterial communities in oyster gills on black tiles were characterised by a significant increase in relative abundance of 117 bacterial Amplicon Sequencing Variants (ASVs), and a decrease in 61 bacterial ASVs (ANCOM‐bc2 P_adj_ < 0.05; Figure 3). Notably, there was a significant increase in the relative abundance of 7 *Vibrio* ASVs when oysters were on the black tiles.

Bacterial communities in oyster haemolymph were characterised by an increase in 165 ASVs, and a decrease of 413 ASVs (ANCOM‐bc2 P_adj_ < 0.05; Figure 3). Again, *Vibrio* ASVs were conspicuous among those ASVs with increased abundance, with five out of the fifty twenty ASVs which increased in abundance assigned to the *Vibrio* genus. ASVs assigned to the genus *Pseudoalteromonas* also occurred in significantly greater abundance in haemolymph from oysters on black tiles (ANCOM‐bc2 P_adj_ < 0.05; Figure 3). ASVs from the genus *Endozoicomonas* decreased in abundance in oyster gill tissues on black tiles, but increased in abundance in oyster haemolymph (ANCOM‐bc2 P_adj_ < 0.05; Figure 3).

Seawater bacterial communities were comprised primarily of SAR 11 bacteria from Clade I, followed by the bacterial families Rhodobacteraceae and Cyanobacteraceae, and *Vibrio* was below 1% of seawater bacterial communities.

### 3.5 Vibrio Community

**FIGURE 4:**
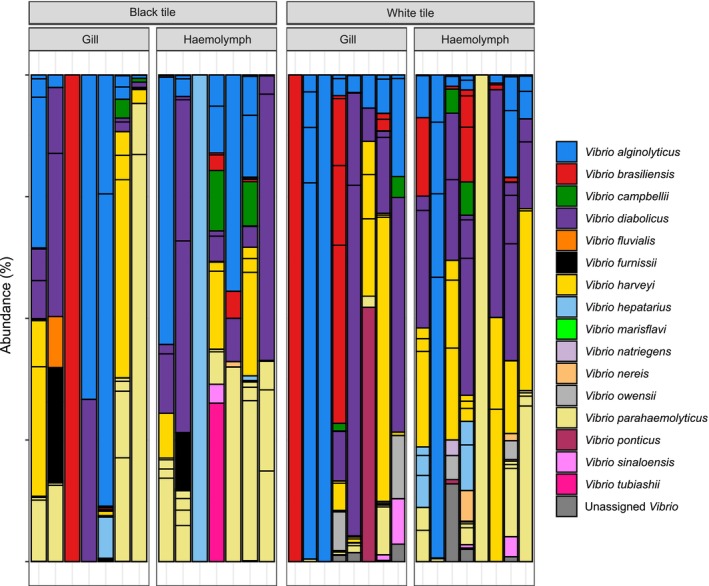
Stacked bar plot indicating the relative abundance of Vibrio bacteria at the species level in oyster gill and haemolymph tissue determined by sequencing of the HSP60 gene.

**FIGURE 5:**
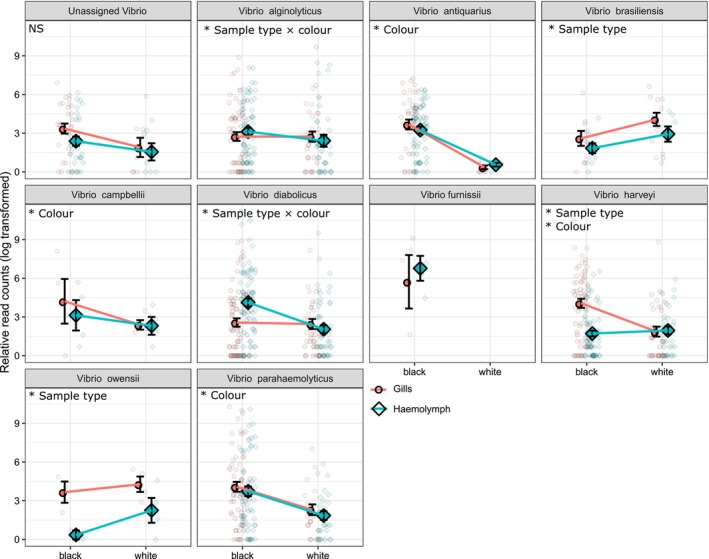
Mean relative abundance of the 10 most abundant Vibrio species (including unassigned Vibrio OTUs) on black and white tiles, and from gill (red circles) and haemolymph (blue diamonds) tissues determined using HSP60 sequencing. Significance as determined by Generalised Linear Mixed Model (GLMM) is indicated with asterisks and text. Small points indicate raw data.

Vibrio communities did not differ significantly between white and black tiles in either gill or haemolymph tissues (PERMANOVA *p* > 0.05; Figure 4) when analysed at the OTU level. However, we did observe significant increases in the relative abundance of some *Vibrio* taxa at the species level (Figure 5). There were significantly greater relative abundances of *Vibrio alginolyticus* (GLMM; Sample type × colour; χ^2^ = 8.2, P_adj_ < 0.05) and *Vibrio diabolicus* (GLMM; Sample type × colour; χ^2^ = 45.9, P_adj_ < 0.05) on black tiles in oyster haemolymph. We also found significantly greater relative abundances of *Vibrio antiquarius* (GLMM; Colour; χ^2^ = 90.5, P_adj_ < 0.05), *Vibrio harveyii* (GLMM; Colour; χ^2^ = 6.8, P_adj_ < 0.05), *Vibrio campbellii* (GLMM; Colour; χ^2^ = 16.9, P_adj_ < 0.05), and *Vibrio parahaemolyticus* (GLMM; Colour; χ^2^ = 68.9, P_adj_ < 0.05) on black tiles in both gill and haemolymph tissues (Figure 5).

## 4 Discussion

Marine and atmospheric heatwaves are potent forces that can reshape ecosystems (White et al. 2023; Oliver et al. 2017). Intertidal habitats are unique in that they are influenced by both marine and atmospheric heatwaves. There is evidence across a wide range of marine organisms that warming can alter host‐microbiome interactions, often reducing host survival and affecting the ecosystems they support (Wernberg et al. 2013; Oliver et al. 2017, 2021). Our novel and simple warming experiment has shown that a small relative increase in temperature can have significant consequences for oyster survival in a real‐world intertidal environment. A modest maximum temperature increase of 3°C and an average of 0.38°C caused a 50% increase in oyster mortality. Accompanying these increases in mortality, we observed a significant shift in the oyster microbiome in both gill and haemolymph tissues. Notably, this microbiome shift involved an increase in both relative and absolute abundance of putative bacterial pathogens from the *Vibrio* genus.

Our results highlight the stresses experienced by sessile organisms in the intertidal zone. Oysters in our experiment experienced increases in temperature from 21.5°C (during emersion at night) to a maximum of 48.5°C on the white tiles over a period of less than 12 h, representing a temperature difference of 27°C. This kind of extreme temperature range has been previously demonstrated in intertidal bivalves (Helmuth, Broitman, et al. 2006). However, our study demonstrated that a further increase in oyster temperature of 3°C was enough to elicit a significant mortality response, a shift in the microbiome and an increase in putative pathogenic microorganisms. Our findings underline the stress and subsequent disease that can occur from intertidal warming, something that was observed on an unprecedented scale in the Northern American heatwave of 2021, where millions of intertidal bivalves were estimated to have died (White et al. 2023).

We observed an altered bacterial community and increased *Vibrio* abundance in the gills and haemolymph of oysters in the elevated temperature treatment. We also saw an increase in *Pseudoalteromonas* in oysters at elevated temperatures. *Pseudoalteromonas* are known to proliferate on dead seafood tissues, associated with seafood spoilage and the storage and transport of dead oysters (Madigan et al. 2014; Chen et al. 2019). We also observed a decrease in *Endozoicomonas* in oyster gills from oysters in the elevated temperature treatment. *Endozoicomonas* are well known to be associated with oyster gills and have previously been found to decrease in relative abundance during times of temperature stress on oysters (Dor‐Roterman et al. 2024). *Vibrio*, however, are known pathogens of oysters and other marine organisms that can cause disease and kill their hosts (Montánchez and Kaberdin 2020; Ina‐Salwany et al. 2019; Zhang and Li 2021). We saw significant increases in the abundance of *V. alginolyticus, V. diabolicus*, *V. antiquarius, V. harveyii, V. campbellii*, and *V. parahaemolyticus* on black tiles. *Vibrio* are common bacteria in coastal environments but can infect marine organisms, especially when their immune system is compromised (De Lorgeril et al. 2018). *Vibrio* produce serine proteases, metalloproteases, and cysteine proteases that cause disease and even mortality in the host when bacteria are in high abundance (Kumar et al. 2021). Interestingly, we observed a significant increase (27 times greater) in the human pathogen *V. parahaemolyticus* in oysters from black tiles. *V. parahaemolyticus* has the ability to cause oyster industry‐wide shutdowns with multi‐million dollar consequences (Taylor et al. 2018). The presence of *V. parahaemolyticus* in these oysters is a concern given elevated *V. parahaemolyticus* abundance in oysters has previously been attributed to elevated seawater temperatures rather than atmospheric temperatures (Scanes et al. 2025; Baker et al. 2022; Baker‐Austin et al. 2013). The ability for atmospheric warming to increase *V. parahaemolyticus* highlights the potential for climate change to disrupt oyster producers around the globe, jeopardising financial and food security.

Our observation that elevated temperatures increased the abundance of putative pathogens from the *Vibrio* genus is consistent with previous studies investigating the microbiome of oysters exposed to elevated temperatures under a number of scenarios (Green et al. 2019; Siboni et al. 2024; Li et al. 2023). For example, two separate experiments examining the impacts of heatwaves under laboratory conditions revealed increases in *Vibrio* abundance in oyster tissues and increased oyster mortality (Green et al. 2019; Scanes et al. 2023). Notably, studies found that antibiotic treatments reduced or eliminated oyster mortality, pointing towards a definitive role of *Vibrio* in oyster mortality (Green et al. 2019; Scanes et al. 2023). Similarly, studies focusing on natural marine heatwaves and seawater warming have demonstrated increased *Vibrio* abundances in oyster tissues (Siboni et al. 2024; Li et al. 2023). Oyster mortality under heat stress is likely caused by compounding physiological and immune factors that allow infection by pathogenic bacteria (Scanes et al. 2023). Our study builds on this body of knowledge by demonstrating this effect in a realistic intertidal experiment.

Our study has highlighted the significance of substrate colour within intertidal shores under high temperature conditions. Naturally darker surfaces in intertidal zones around the globe are likely to become even hotter under climate change and reshape the communities that colonise them. Rocky shores possess microclimates and habitat refugia that will likely become even more valuable as climate warming continues and heatwaves become more frequent and intense (Helmuth, Mieszkowska, et al. 2006; Helmuth, Broitman, et al. 2006). Indeed, we found that natural oyster aggregations reached a maximum temperature 3.5°C cooler than that reached on the white tiles, likely due to the tiles horizontal surface and the three‐dimensional shape of natural aggregations trapping water in the shell matrix (Helmuth and Hofmann 2001).

Intertidal habitats are also vulnerable to stressors from the ocean as well as the atmosphere. The shallow nature of many estuaries in Australia and around the globe will lead to estuarine water temperatures rising faster than the open ocean (Scanes, Scanes, and Ross 2020). Such extreme shallow water warming is likely to promote *Vibrio* growth (Baker‐Austin et al. 2017). This has been observed during summer in shallow estuarine mudflats in Oregon, USA, where *Vibrio* reached concentrations 1000 times greater than local coastal seawater (Gradoville et al. 2018). This indicates that intertidal organisms will be exposed to greater pathogen loads when immersed at high tide.

Our findings are relevant for intertidal habitats globally. Already, intertidal organisms are living close to their physiological limits (Helmuth, Mieszkowska, et al. 2006). As a result, a relatively small increase in temperature can push them over a threshold of survival and alter host–microbiome interactions, with conditions often tipping in the favour of microbial pathogens. The Earth's climate is predicted to warm further in the coming decades regardless of global emissions reductions (Cooley et al. 2022). Australia's atmosphere has already warmed by 1.47°C since 1910 and is expected to reach 3°C warming by 2040–2050 (Bureau of Meteorology 2018). Our findings demonstrate that an increase of 3°C in a real‐world experiment can shift the oyster microbiome and significantly decrease oyster survival. Therefore, we posit that near‐future warming is likely to place further selective pressure on oyster populations around the globe, triggering them to adapt or alter their distribution. These changes will likely come with consequences for the ecosystems they support and aquaculture production.

## Author Contributions

**Elliot Scanes:** conceptualisation, methodology, investigation, writing – original draft, writing – review and editing, resources, supervision, formal analysis. **Nachshon Siboni:** methodology, investigation, writing – review and editing, resources, data curation. **Maquel Brandimarti:** conceptualisation, methodology, writing – original draft, writing – review and editing. **Justin Seymour:** methodology, writing – review and editing, resources.

## Conflicts of Interest

The authors declare no conflicts of interest.

## Data Availability

All genetic sequences will be available at the NCBI sequence archive (SRA). All other data is available on request.
